# Relative Roles of Race Versus Socioeconomic Position in Studies of Health Inequalities: A Matter of Interpretation

**DOI:** 10.1146/annurev-publhealth-040617-014230

**Published:** 2018-01-12

**Authors:** Amani M. Nuru-Jeter, Elizabeth K. Michaels, Marilyn D. Thomas, Alexis N. Reeves, Roland J. Thorpe, Thomas A. LaVeist

**Affiliations:** 1 Division of Community Health Sciences, School of Public Health, University of California, Berkeley, California 94720, USA; 2 Division of Epidemiology, School of Public Health, University of California, Berkeley, California 94720, USA; 3 Department of Health, Behavior, and Society, Bloomberg School of Public Health, Johns Hopkins University, Baltimore, Maryland 21205, USA; 4 Department of Health Policy and Management, Milken Institute School of Public Health, George Washington University, Washington, DC 20052, USA

**Keywords:** race, socioeconomic position, socioeconomic status, health inequalities, social epidemiology, social determinants of health

## Abstract

An abundance of research has documented health inequalities by race and socioeconomic position (SEP) in the United States. However, conceptual and methodological challenges complicate the interpretation of study findings, thereby limiting progress in understanding health inequalities and in achieving health equity. Fundamental to these challenges is a lack of clarity about what race is and the implications of that ambiguity for scientific inquiry. Additionally, there is wide variability in how SEP is conceptualized and measured, resulting in a lack of comparability across studies and significant misclassification of risk. The objectives of this review are to synthesize the literature regarding common approaches to examining race and SEP health inequalities and to discuss the conceptual and methodological challenges associated with how race and SEP have been employed in public health research. Addressing health inequalities has become increasingly important as the United States trends toward becoming a majority-minority nation. Recommendations for future research are presented.

## 1. INTRODUCTION

Several papers (empirical and review) have documented health inequalities by socioeconomic position (SEP) and race in the United States. The public health literature consistently demonstrates that (*a*) racial inequalities exist across health outcomes, (*b*) SEP inequalities exist across health outcomes, (*c*) SEP attenuates racial inequalities across health outcomes, and (*d*) there are residual effects of race on health after controlling for a variety of socioeconomic indicators. Together these studies show that race and SEP explain both unique and shared variance in relation to a wide variety of health outcomes. As a result, the inclusion of race and/or various indicators of SEP as either control variables or effect modifiers has become a habitual, and often atheoretical, practice in epidemiologic studies (59). In contrast, some studies use one or the other as primary exposures with the goal of investigating how the social meaning, and hence positioning, of individuals and groups in society based on race and/or SEP determines health inequities. However, this approach may also, often and likely unintentionally, reify social stratification (present company not excluded) through the at times problematic use of social statistics, the statistical analysis of social (e.g., racial) classification (see the sidebar titled Social Statistics) (128).

In this article, we review the most commonly used approaches to examining race and SEP health inequalities in the public health literature and discuss what we see as some of the most significant conceptual and hence methodological challenges associated with, or that complicate, interpreting this literature. We then reflect on the critical issue of the ways that race has been defined in US society—using examples of Blacks and Whites—to expose the vulnerability of public health research to inadvertently reinforcing and perpetuating health inequities by uncritical adoption of current conventions in the measurement and interpretation of race differentials, as such and particularly with regard to SEP inequalities. We conclude with recommendations for new directions in this domain of public health research.

## 2. COMMON APPROACHES AND METHODOLOGICAL CHALLENGES

Common applications of race and SEP as variables in public health research include their use as (*a*) surveillance variables, (*b*) control variables, and (*c*) primary exposures, as well as efforts to model both their (*d*) unique and (*e*) joint effects in relation to health outcomes. Below, we describe the utility, conceptual assumptions, and methodological challenges associated with these common uses of race and SEP, with a focus on issues related to measurement, causality, and validity.

### 2.1. Race and SEP as Surveillance Variables

Public health surveillance efforts in the United States, including those conducted by the Centers for Disease Control and Prevention and the US Census Bureau, among others (23, 55, 71, 86), commonly disaggregate reports by sex and by race. In contrast with the United Kingdom, where class-based gradients in health have been monitored for decades (43, 84), surveillance efforts in the United States have been less consistent in reporting health statistics by SEP (3, 23, 59, 74). In part, this practice reflects variations in data collection practices between the two countries. According to some, it also demonstrates the conceptual assumption that race is the most meaningful category upon which data should be collected and stratified in the United States (59). Notably, the US Affordable Care Act mandates data collection by race but not by SEP (90). As we discuss below, race is often used as a proxy for SEP, a practice that may reflect the limited nationally representative data available for SEP. Limitations notwithstanding, numerous studies have demonstrated striking variations in health along both racial and socioeconomic lines, underscoring the significance of these characteristics as enduring factors by which health outcomes are patterned (2, 16, 43, 78, 83, 84).

Descriptive (i.e., surveillance) reports of health inequalities are useful for raising professional and public awareness of racial and socioeconomic health inequalities, demonstrating trends in these inequalities, prioritizing funding and interventions, generating hypotheses, and informing more focused research efforts (40). However, they do not provide insight into why these inequalities exist. If not interrogated further, purely descriptive data may reify race as a biologic category and affirm social stratification as a normative aspect of civil society, leading to the conclusion that observed inequalities are simply the nature of things (59). Framed differently, many observed racial and socioeconomic differences in health may be viewed as inequities, which by definition are unjust and preventable (15, 90). If, then, the goal is to interrogate and intervene on racial and socioeconomic health inequities, purely descriptive data will not suffice. Successful program and policy interventions require evidence on the root causes of and mechanisms through which health inequities persist. In support of this goal, a substantial body of literature has explored both the independent and joint effects of race and SEP as determinants of health. In Sections 2.3–2.5, we describe common approaches, conceptual considerations, and methodological challenges associated with assessing race and SEP as determinants of health. First, we discuss a more common strategy: their use as control variables.

### 2.2. Race and SEP as Control Variables

**Figure 1:**
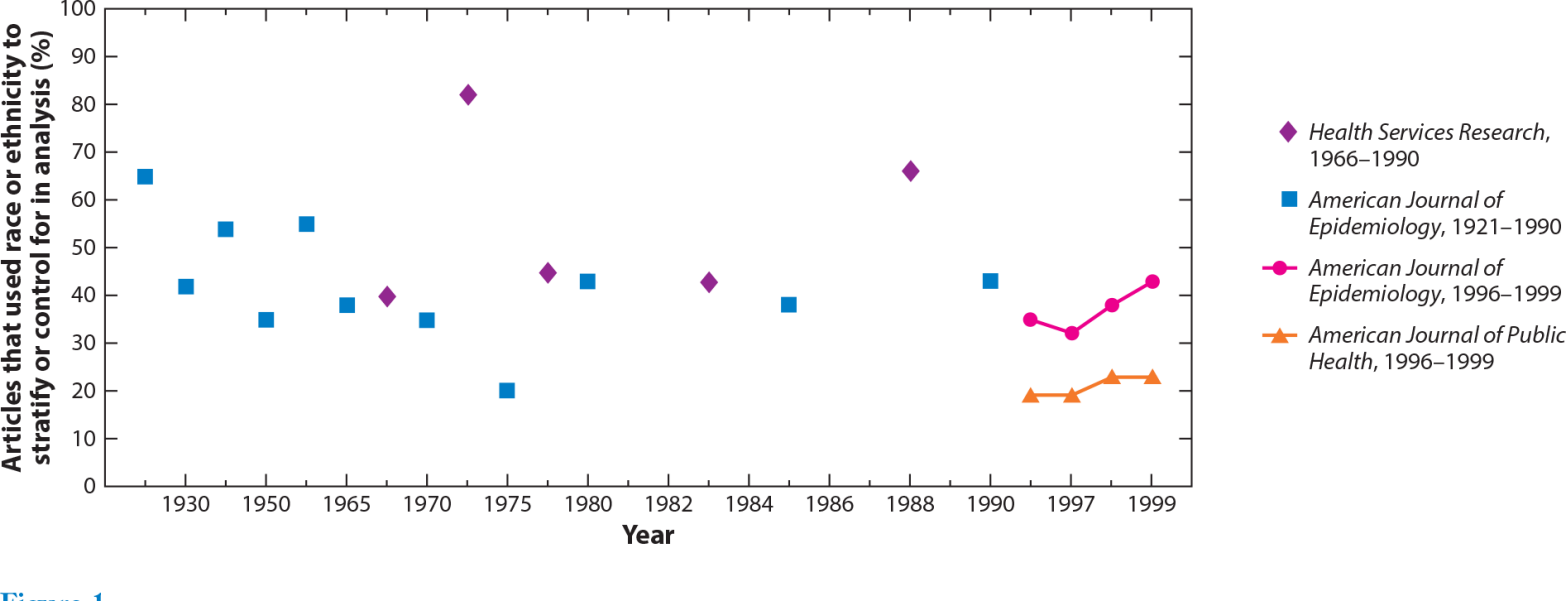
Comparison of the proportion of journal articles that used race or ethnicity to stratify or adjust for in analysis. Adapted with permission from Reference 32, *American Journal of Epidemiology* © 2004; 159(6):611–9. Comstock RD et al. Four-year review of the use of race and ethnicity in epidemiologic and public health research. Published by Oxford University Press. Printed with permission. All rights reserved.

**Figure 2:**
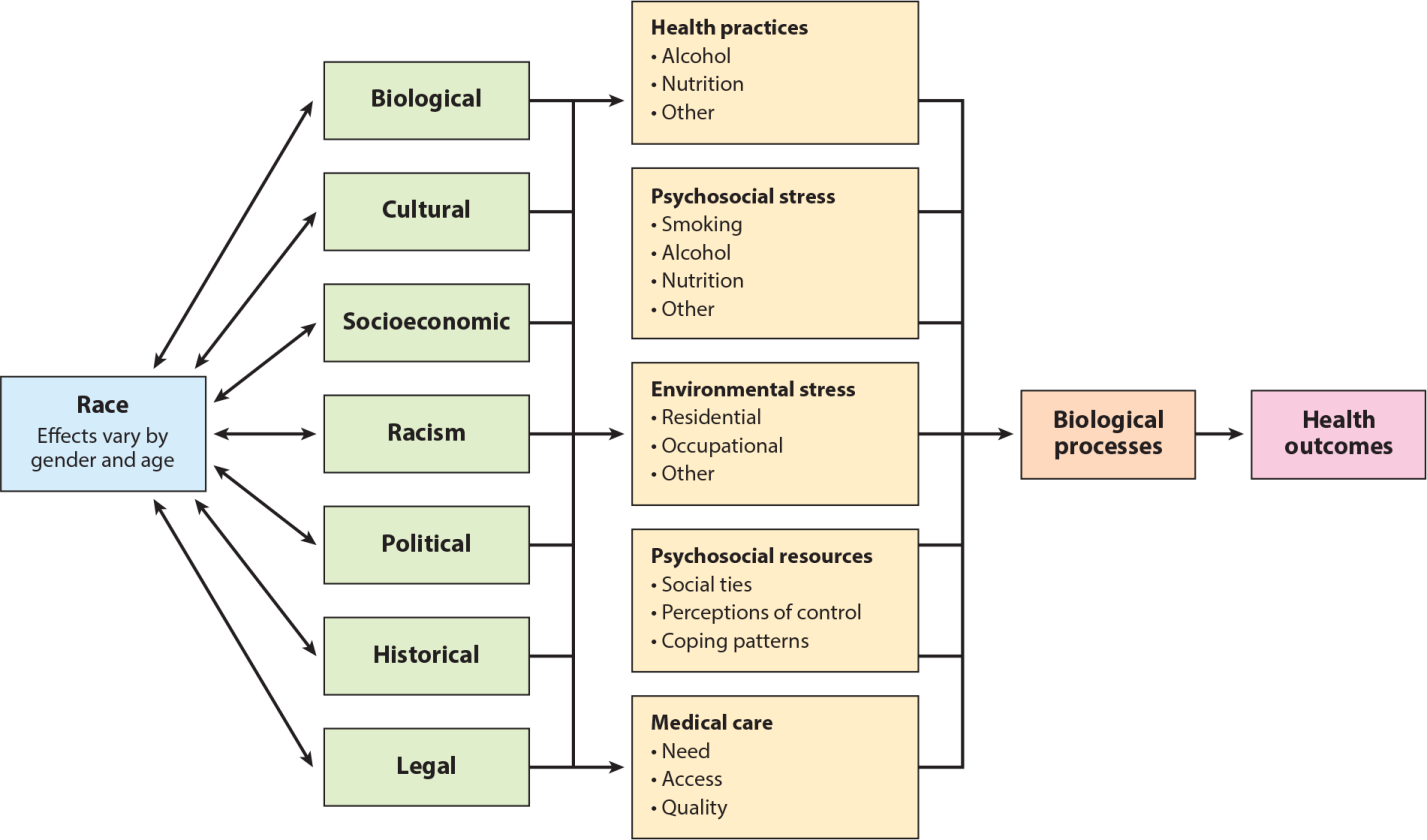
A framework for understanding the relationship between race and health. Adapted with permission from Reference 23, *Summary of the CDC/ATSDR Workshop* © 1993. Use of race and ethnicity in public health surveillance. Published by Prevention at the Centers for Disease Control. All rights reserved.

Given the pervasive nature of racial and socioeconomic health inequalities, race and/or SEP are commonly included as covariates (i.e., control variables) in the public health literature (32, 45, 59, 64, 68, 110). More than 25 years ago, Jones et al. (57) published the first review of how race and ethnicity are used in the epidemiologic literature, covering the period 1921–1990, and made recommendations for improving understanding of root causes of racial health inequalities. Since that time, a number of similar reviews have been published, many with recommendations on improving the use of race in public health research (4, 23, 32, 80, 119). In one of the most recent investigations, Comstock et al. reviewed 1,098 articles published in the *American Journal of Epidemiology* and the *American Journal of Public Health* from 1996 to 1999 and found that the percent of articles using race and/or ethnicity as control variables has shown an upward trend compared with earlier time periods, where controlling for race was relatively stable over time (Figure 1) (32). In their review, the most common practice was to control or adjust for race. Notably, 57% of articles did not state their purpose for including race as an analytic variable. The primary use of race as a control variable belies broader conceptualizations of how health risk is patterned by race (Figure 2) (23).

SEP measures are also commonly used as control variables, presenting similar challenges in interpreting study findings (40). To examine current trends in the use of race and SEP in public health research, we reviewed articles published over the past four years in the *American Journal of Epidemiology* (*AJE*) and the *American Journal of Public Health* (*AJPH*) (2013–2016). Of the 836 empirical studies published in the *AJE*, 515 (62%) included race and/or SEP as control variables, with only one-third providing an explicit rationale for their use. Notably, the percent including race and/or SEP as controls has increased over time, from 52% in 2013 to 82% in 2016. On the contrary, the number of studies including a rationale for adjusting for race and/or SEP has declined from 39% in 2013 to 22% in 2016. Similarly, of the 1,200 empirical articles published in the *AJPH* over the same time period, 67% (*n* = 799) included race and/or SEP as control variables, whereas only 33%(*n* = 266) included an explicit rationale for their use. Notably, a review of article citations in the *AJE* from 1981 to 2002 found that only a minority (0.2%) were from the social sciences (Supplemental Figure 1) (94), potentially contributing to the overuse of race and SEP as controls.

Although the reasons for adjusting for race and SEP are not always clear (32, 59), the tacit assumption is that these factors are confounders that must be controlled for to isolate the exposure–outcome relationship of interest (16, 32, 45, 59, 64, 68, 78, 110). Critiques of the adjustment for race and SEP include (*a*) lack of conceptual and hence operational specificity in measurement (variable selection and specification) and proposed pathways to health, (*b*) masking racial and SEP inequities in the distribution of exposures and outcomes, and (*c*) inattention to the ways in which race and SEP may modify the association between the exposure and outcome of interest.

First, in relation to variable selection and specfication, Braveman et al. (14) examined how adjusting for different measures and specifications of SEP influence the conclusions drawn about racial inequalities in maternal and infant health outcomes among a representative sample of postpartum women in California. Each model consisted of a different specification of either income or education, the two most commonly used SEP indicators in public health research. For a given health outcome, whether there was a statistically significant racial inequality and how strong it was varied depending on both the particular SEP measure (i.e., income or education) and how the measure was specified (e.g., continuous versus categorical and if categorical how categories were defined), demonstrating the importance of variable selection and specification for the validity of study findings. Furthermore, for a given operationalization of SEP, results varied across racial groups and were dependent on the health outcome under investigation. Braveman et al. (14) concluded that SEP measures should be chosen and specified on the basis of the proposed pathways to health for a given racial group. Others have similarly concluded that SEP may impact health via different mechanisms depending on how it is conceptualized and measured (9, 14, 16, 76). However, most studies rely on only a subset of socioeconomic indicators, potentially resulting in residual confounding, which may bias effect estimates (16, 65, 88).

Second, controlling for race and/or SEP when attempting to isolate another exposure–outcome relationship of interest artificially creates equality in both the exposure and the outcome along the dimensions of race and SEP, obscuring potentially meaningful information about the mechanisms underpinning health inequities (64, 78), and compromises the ecological validity of study findings (16, 65, 67, 78, 87). Hence, adjustment for race and SEP has been criticized for treating these factors as “nuisance confounders rather than important clues to be mined” (59, p. 302). Finally, ignoring potential effect modification can (*a*) reduce the precision of effect estimates diminishing opportunities for targeted intervention (64, 78), (*b*) mask the true nature of risk in the sample/population, and (*c*) hinder the ability to interrogate interactions that could illuminate mechanisms through which health inequities are produced and maintained.

### 2.3. Race and SEP as Exposures

Much of contemporary social epidemiology has moved beyond simply describing or controlling for differences in health by race and SEP, focusing instead on understanding the mechanisms by which inequities are produced and sustained. In pursuit of this charge, social epidemiologists often conceptualize and model race and/or SEP as primary exposures (20, 41, 50, 51, 81, 95, 103, 112). Interpreting these coefficients, however, requires clear conceptual and operational definitions of these constructs, a task that is complicated by their multifactorial nature and data source limitations (16, 59).

#### 2.3.1. Race as an exposure.

Although not always explicitly stated, estimating the effect of an exposure (e.g., a race effect) on a given outcome relies on causal models and is thus subject to their assumptions and limitations (64). A distinct feature of causal inference is the articulation of a counterfactual quantity (106, 108, 109). Articulating this quantity requires imagining a hypothetical experiment or intervention to change treatment or exposure status. Specifying a counterfactual for race has been the subject of several lively point–counterpoints over the past several decades (34, 42, 61–63, 73, 89, 118). Rather than rehashing these arguments, we contend that this debate relates back to the fundamental question: What is race?

The pan-ethnic categories utilized by the Office of Management and Budget obscure considerable within-group genetic/biologic, social, and cultural heterogeneity (11, 85, 122), potentially threatening the ability to make reliable inferences (59, 64). When assumptions about the meaning of race are not made explicit (e.g., social versus biological), the effect estimate produced leaves much to the imagination. Even when assumptions are made explicit, it is questionable whether valid causal conclusions can be drawn. When a race effect is observed, and particularly when this effect persists after adjusting for socioeconomic factors, researchers may rely on a priori genetic/biologic, sociopolitical, and/or cultural assumptions about the meaning of race. Thus, getting the counterfactual right requires carefully specifying the proposed causal component and measuring it explicitly (10).

In contrast with the historically dominant understanding of race as a biologic category (33), many view race as a social-contextual and relational construct shaped by systems of power and privilege—i.e., racism (59, 23, 124). The latter emphasizes that racial categories, and the meanings ascribed to them, are socially produced and vary across time and place (59). Conceptualizing race in this way, rather than as an essential genetic/biologic attribute, is helpful for defining both the factual and the counterfactual quantities of interest, which are often not racial groups per se but rather factors that create and maintain racial health inequities (e.g., structural, personally mediated, and internalized racism) (58). Within-group study designs allow for an assessment of heterogeneity within racial groups (38), thus avoiding the use of averages to describe differences between racial groups and providing a more appropriate counterfactual quantity (30, 59). Thus, although race may be useful for describing inequalities between racial groups (i.e., surveillance), complications arise when the research question pertains to why inequalities exist and causation is inferred.

#### 2.3.2. SEP as an exposure.

SEP is one of the most widely recognized and enduring predictors of population health (43, 53, 83, 84). Although various indicators of SEP have been consistently associated with numerous health outcomes, inferring causality on the basis of observed associations is threatened by (*a*) reverse causation and confounding, (*b*) inconsistencies in the conceptualization and measurement of SEP, (*c*) data limitations, and (*d*) violation of stability assumptions (1, 3, 8, 14, 43, 66, 100). First, some researchers have suggested that having poor health causes lower income and less education (i.e., social selection), rather than the other way around (i.e., social causation), or that the two exist in a reciprocal relationship (70, 113). Although the weight of the evidence is in favor of social causation, failure to establish temporality threatens causal inference (3, 68). Furthermore, associations may vary for different health outcomes (14, 15). Although establishing socioeconomic factors as causal is challenging in observational studies, quasi-experimental design, natural experiments, and longitudinal studies have been proposed as promising alternatives (10, 66).

Second, SEP is a multidimensional construct that may affect health via different mechanisms, at multiple levels, across the life course, and differentially for various population subgroups (3, 14, 16). Current measures of SEP build on Karl Marx’s materialist theory of social class and Weber’s multidimensional theory of stratification, which conceptualizes both material and nonmaterial sources of status and power (76). Variables may include absolute measures capturing ownership of material resources (e.g., income, wealth, employment status) and/or relative measures capturing rank or prestige (e.g., occupation, level of educational attainment, subjective social status) (76). However, as described above, these indicators are generally only moderately correlated and relate to different pathways to health (14). Additionally, their effects may vary depending on the particular health outcome and/or study population under investigation (14). Some studies include individual-level measures as well as area- or group-based measures, and the interaction between the two (76), which minimizes measurement error and provides a more comprehensive assessment of socioeconomic context. Although some consider education to be perhaps the most stable indicator of SEP and therefore the least susceptible to reverse causality (17), several excellent reviews have described the importance of choosing measures that are consistent with proposed pathways to health (16). Last, assessing SEP over the life course with attention to change over time (e.g., patterns of mobility) addresses challenges associated with social selection due to socioeconomic histories and the resulting shift in life course health trajectories prior to the time of observation (Supplemental Figure 2) (67, 76). Despite this complexity, SEP is generally measured at one point in time using various specifications of income, education, occupation, or some combination thereof (14, 63). Many studies rely on just one of these indicators, risking measurement error and biased effect estimates due to residual confounding (65).

Another important, albeit less commonly studied, measure of SEP is wealth, described as “total accumulated economic resources” [e.g., real estate, account holdings (16, p. 2883)]. Wealth may buffer the deleterious health consequences of periods of low income (16, 76). Wealth is important for life planning, attaining and maintaining prestige, and broadening social networks and is a financial guarantee for both present and future generations. Although income, occupation, and education are the most commonly used indicators of SEP, they are imperfect proxies for wealth (97), especially in relation to racial inequalities. Within income strata, studies show substantial inequalities in net worth across racial groups. Racial minorities have just a fraction of the net worth (total accumulated assets) and net financial assets (accumulated nonphysical/liquid assets) as do Whites, inequalities that are masked when measuring only income (Table 1) (77, 79, 121). Notably, the racial wealth gap has been remarkably stable over time (96). Studies have consistently demonstrated that income is a stronger predictor of health than is race (96) because income differences in health within a given racial group are often much greater than racial differences within income strata (120). However, income greatly underestimates socioeconomic differences between racial groups, resulting in significant misclassification of risk.

Data collection presents another methodological challenge related to the measurement of SEP, particularly measures of income. Income categories are often capped at levels that preclude an examination of the highest income groups, masking heterogeneity and potentially meaningful information about associations between income and health for middle- and upper-socioeconomic groups (120), where some of the greatest racial health inequalities have been found (18, 21, 41, 100, 123, 126). For example, income is capped at ≥$84,000 in the National Health and Nutrition Examination Survey, ≥$75,000 in the National Health Interview Survey, ≥$50,000 in the Behavior Risk Factor and Surveillance Survey, and ≥$25,000 in the General Social Survey (24, 25, 37). However, studies show racial health inequalities to be greatest at higher versus lower income levels. One recent study examined racial health inequalities among those with a reported income ≥$175,000 and found a significant health disadvantage among African Americans relative to other groups (126). Others have similarly found that increasing income is not protective for all groups (90), a difference that is masked when income is capped at lower levels, again resulting in misclassification of risk.

Similarly, education is most often assessed either by years of schooling completed or by highest credential earned (14). Both fail to capture the quality of education received, which varies considerably depending on geographic region, school district, and funding resources. Many Black and Latina/o children receive a lower-quality education than do their White counterparts, likely contributing to income inequalities at similar education levels later in life (14, 56). Hence, similar levels of income or education do not buy the same social, economic, and health gains for all groups (100).

### 2.4. Modeling the Unique Effect of Race and SEP

The high degree of confounding between race and SEP has motivated an extensive literature seeking to disentangle these two social determinants of health (79, 101, 123, 124). Studies often statistically adjust for SEP to isolate the unique (i.e., unconfounded) effect of race on a given health outcome (12, 20, 41, 42, 50, 51, 59, 64, 81, 103, 112, 118). Similarly, albeit less commonly, efforts to isolate the independent effect of SEP on health involve statistically adjusting for race (20, 95). The conceptual and methodological considerations when modeling the unique effect of SEP, adjusting for race, have been described in previous sections (Sections 2.2 and 2.3.2). Hence, we turn our attention to the more common approach of modeling the unique effect of race (14, 20, 41, 42, 50, 51, 59, 64, 81, 103, 112, 117) while adjusting for SEP from the perspective of two common strategies: mediation and moderation.

#### 2.4.1. Mediation.

Kaufman & Cooper (64) have argued that “a person’s race is fixed prior to his/her measured social, physiologic, and psychological status; all of these factors are downstream of the exposure in a racially stratified society” (p. 294). Thus, adjusting for these factors may result in overcontrolling, potentially threatening the validity of the race effect (Supplemental Figure 3) (33, 64, 65, 89).

Although more sophisticated models are emerging (7), the traditional mediation models described in this section generally assume no statistical interactions between the primary exposure variable and the intermediary (64, 88). If SEP is conceptualized as a confounder, the residual race effect will be interpreted as the race effect not confounded by SEP (118). As described above, this interpretation may overestimate the race effect owing to residual confounding by unmeasured or misspecified SEP factors or may underestimate the race effect by not accounting for its indirect effects mediated by SEP (33, 64, 65, 88). If SEP is conceptualized as a mediator, the residual race effect can be interpreted as the direct effect, unmediated through SEP (64, 118).

**Figure 3:**
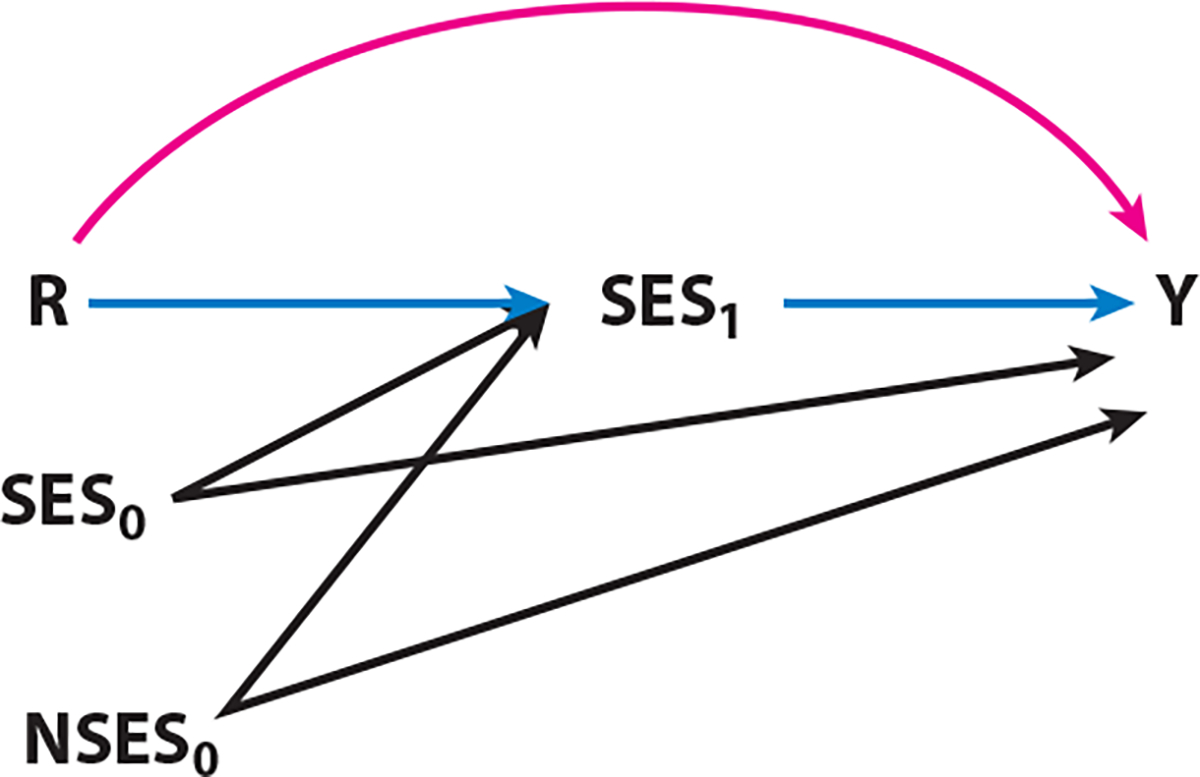
Time-dependent socioeconomic status pathways over the life course. Direct effects of race (R) on health (Y) (*the pink pathway*) and indirect effects through adult SES (*the blue pathways*), where both SES_0_ (family/parent SES) and NSES_0_ (neighborhood socioeconomic status) contribute to adult SES. Adapted with permission from *Epidemiology* © 2014; 25(4):473. VanderWeele TJ, Robinson WR. On the causal interpretation of race in regressions adjusting for confounding and mediating variables. Published by Wolters Kluwer Health, Inc. Printed with permission. All rights reserved. http://journals.lww.com/epidem/pages/default.aspx

The distinction between SEP as a confounder versus as a mediator may depend on the timing of its measurement (118). SEP at birth may confound the race effect due to prior social and historical processes (118). However, when SEP is measured in adulthood, as it frequently is, its mediating role becomes clearer. In this case, “the overall racial inequality can be decomposed into the portion that would be eliminated by equalizing adult SEP across racial groups and the portion of the inequality that would remain even if adult SEP across racial groups were equalized” (Figure 3) (118, p. 474). Because SEP is hypothetically more manipulable than race (as opposed to particular social experiences such as racial discrimination), this interpretation helps to ameliorate some of the counterfactual critiques raised previously (34, 63, 89). This approach, of course, requires that relevant pathways to health are conceptualized and measured appropriately.

As we have discussed, racism, rather than race, has been proposed as a more salient quantity of interest in explaining racial health inequities (28, 58, 59, 101, 124, 125). Racism affects health by structuring the distribution of socioeconomic resources between racial groups, as well as through nonmaterial mechanisms such as psychosocial stress and hence has been proposed as a fundamental cause of health (101). Phelan & Link argue that (*a*) racism is a fundamental cause of racial differences in SEP; (*b*) SEP is a fundamental cause of inequalities in health and mortality (*a* + *b* = indirect effect of racism); and (*c*) racism is a fundamental cause of racial differences in health and mortality independent of SEP (direct effect) (101, p. 313) (Supplemental Figure 4). However, when race (versus racism) is conceptualized as the exposure of interest, the direct effect (represented by the race coefficient) becomes difficult to interpret, again leaving interpretation to the imagination. As we have discussed, bias may be introduced when researchers make conjectures about which unmeasured correlates of race explain the residual race effect estimate (33, 59, 65, 88).

#### 2.4.2. Moderation.

A mounting body of evidence demonstrates intersections between race and SEP on health outcomes (5, 21, 36, 41, 54, 75, 123). For example, significant racial health inequalities exist at every level of SEP (75, 123) and may be particularly pronounced at very high levels of income, wealth, and education (14, 20, 41, 123, 126). Similarly, associations between SEP and health may differ by race (111, 115), underscoring the importance of examining the effects of one by levels of the other. Investigating differences by race also raises the important issue of our preoccupation in public health with modeling “average” effects. The most common strategies used (i.e., descriptive/surveillance variables and control variables) rely on methods that assess the average health response for the average African American, Asian, Latina/o, Native American, and White person and for a person with average income or education. Not only are there problems with assessing the average Asian or Latina/o, for example—what does that mean given that each of those groups represents a panethnicity?—but even when disaggregated into more appropriate subpopulations, assessing the average health response for the average Cuban or Filipina/o masks the tremendous heterogeneity within each of those subgroups with respect to both exposure and outcome. Some of the less commonly used methods such as random forests start to address this challenge by using decision rules based on how often a random individual would be misclassified to optimize classification of all individuals rather than the average individual (49). Similarly, regularization methods (e.g., ridge regression and Lasso) are conceptually better aligned with balancing the existing benefits of regression with qualitative objectives (49). The use of such techniques allows for optimal selection of variables in explaining a certain outcome, given a set of data (49).

Finally, the stability assumption is often violated in efforts to isolate the unique race effect because measures of SEP are incommensurable across racial groups, as described above (16, 100, 125). Thus, models that control for SEP to isolate a race effect may not only violate stability assumptions but also ignore potentially salient interactions between race and SEP in the production of health inequity (see the sidebar titled Diminishing Returns Hypothesis).

### 2.5. Shared Variance: A Way Forward

The conceptual and methodological challenges associated with isolating the independent effects of race and/or SEP on health, coupled with a large body of literature demonstrating intersections between the two, support the need to consider shared variance associated with race and SEP for health outcomes—the ways in which the two operate synergistically to impact health. We describe two promising approaches: intersectionality and place-based interactions.

#### 2.5.1. Intersectionality.

A growing body of public health literature is applying an intersectional framework to investigate the synergistic effects of multiple axes of social disadvantage. This, scholars suggest, is a more ecologically valid approach to understanding lived and social experiences associated with interlocking systems of social oppression (8, 13, 31, 46, 125). Intersectionality considers how people experiencing multiple social inequalities simultaneously experience excess risk—risk greater than the sum of each individual risk (46, 54, 69). Although qualitative methods have been the gold standard for investigating intersectionality given their search for deep meaning (13), quantitative public health scholars are becoming more explicit in their attempts to investigate health risks associated with multiple marginalized social identities (54), what some have referred to as multiple jeopardy (69, 93). Thus, mixed methods studies assessing intersectionality are well suited for public health research, particularly work that incorporates social theory aimed at elucidating (i.e., contextualizing) the “social-psychobiological” (27) mechanisms by which the experiences of intersecting social identities are embodied (8, 46, 54, 72), a “causes-of-effects” approach (82, p. 230). Mixed methods can enrich understanding of intersectionality in relation to health by interrogating the unique experiences of different groups, thereby aiding in more informed research questions and hypotheses, more creative study designs, and more appropriate data collection instruments. This approach goes beyond simply studying intersectional identities (e.g., Black middle-class heterosexual man) and requires attention to social processes (e.g., discrimination, relational expressions of masculinity among racially oppressed groups) at multiple levels (i.e., individual, interpersonal, institutional, structural) (13). Additionally, intersectionality emphasizes heterogeneity within and across social categories, motivating investigators to revisit previous assumptions and conceptualizations of race and SEP to improve the validity of health inequalities research. We previously found that additive models mask important health differences between groups defined simultaneously by race, gender, and income, demonstrating how modeling decisions impact the validity of study findings (Supplemental Table 1) (93). Recent studies have similarly showed striking differences when comparing additive to multiplicative models (54). As public health scholars answer the call to move beyond asking “race or class” and instead ask how “race *and* class” pattern health inequities (92), further work will be needed to understand the strengths and limitations of various methodological approaches (8). Studies making explicit use of intersectionality conceptually and methodologically are rare in public health but are a promising way forward in understanding and ameliorating health inequities.

#### 2.5.2. Place-based determinants.

The United States is segregated by race (79) and, to a lesser degree, income, creating different exposures to economic opportunity and other community resources that enhance health as well as a host of social and environmental risks (77, 121). Thus, place is confounded by race. The Exploring Health Disparities in Integrated Communities (EHDIC) study represents a novel approach to investigating the synergistic effects of race and SEP. The EHDIC study was designed to compare the health of Blacks and Whites of the same individual-level SEP who live in racially integrated communities and are therefore exposed to the same set of social and environmental conditions (77). This approach affords the opportunity to examine the shared variance explained by both individual- and area-level SEP and race. Two contiguous census tracts in Southwest Baltimore, Maryland, containing ≥35% Black and ≥35% White residents with a Black-to-White median income ratio and high school graduation rate ratio between 0.85 and 1.15 were identified. Trained interviewers administered structured in-person interviews with adult residents aged 18 and older. Blood pressure was measured using standard procedures. The study questionnaire incorporated questions from three national surveys in order to compare results from analyses of national samples, which do not account for segregation, with the two racially integrated communities from Southwest Baltimore. Study findings show that “there were, in fact, no disparities in health status by race because both Blacks and Whites were experiencing the same high rates of adverse health events” (90, p. 60). The study authors concluded, “Race is not protective if you live in an environment that is going to produce bad health outcomes” (p. 60), demonstrating the ways in which place and race intersect to determine patterns of health outcomes between groups.

## 3. ARRIVING AT ACCEPTABLE CONCEPTUALIZATIONS OF RACE

The definition, conceptualization, and operationalization of race and SEP are fundamental to understandings of how these constructs combine to pattern health. We have discussed many of the challenges around operationalizing SEP in public health research, advocating for careful variable selection and specification to avoid measurement bias and residual confounding. As discussed above (Section 2.3.1), the biological basis versus social construction of race poses a fundamental point of departure and source of ongoing debate in research seeking to explain racial health inequities. Adding to this lack of clarity, studies almost never indicate their particular definitions or conceptual basis of race, leaving the interpretation of study findings to one’s imagination with inherent biases depending on one’s ontological views and interpretive frameworks (e.g., positivist, constructivist, critical race theory) (35, 82). Although acknowledging one’s philosophical and interpretive frame is a staple of qualitative research, quantitative studies rarely report their ontological and epistemological stance or their particular interpretive frame. However, as Yanow & Schwartz state, “Interpretive work entails a ‘philosophical rigor’—a rigor of logic and argumentation—rather than merely a procedural ‘rigor’” (127, p. xix). Others have expressed concerns about epidemiologic studies as “instruments of ‘decontextualization’” (107, p. 811) and as “inappropriate in studies that require a consideration of historical and social context. The danger is that attempting to eliminate the influence of all other causes of diseases—in an attempt to control confounding—strips away the essential historical and social context” (99, p. 682).

Here, we consider how our conceptual and operational definitions determine our use of social statistics to potentially reify social stratification (128). Often used as a catch-all category, the uncritical use of race as a variable in health research constrains deeper reflection on the meaning of race for determining patterns of population health and, as stated previously, limits our pursuit of identifying a proper counterfactual quantity. Hence, we return to the question, what is race? The question has been debated for years. Whereas some define race as a social construction, others define race as biological. Each view has its history, the former in relation to the process of colonization and slavery for economic gains during the western spread of the sugar industry and eventual expansion to the United States (128). This process of economic and racial subjugation laid the foundation for first de facto and then de jure racial classification in the United States, the remnants of which are present to this day despite the cloak of color-blind racism, the myth of meritocracy, and postracial politics (96, 98), all of which have been challenged in the present Trump era (29). At issue is the process and experience of social stratification and the ways in which one’s location in the social structure determines access to resources (physical, social, political, and economic). Recent studies show that being socially assigned as a racial minority versus White, regardless of self-identified race, is associated with a significant health disadvantage; some studies suggest that this association is partially due to experiences of racial discrimination and socioeconomic hardship (Supplemental Figures 5–7) (48, 60, 78). Studies also show that racial health inequalities persist across socioeconomic strata, including among those with at least four times the national median income (90, 126). These findings illustrate the synergistic role of race and SEP as well as unique aspects of the social experience of race that contribute to health inequities. The latter definition of race (race as biological), though having its roots in eugenics, continues to find a home in biomedical discourse. Quoting Zuberi (128),

The history of social statistics reveals the ambiguities that underlie racial statistics and remind us how our racial concepts have influenced the logic of statistical methods. The population perspective in both demography and statistics corresponds to the tendencies of group objectification in social statistics…. This perspective views groups as entities with collective traits that can be statistically described. (pp. 29–30)

As an example of this ambiguity, the most recent dictionary of epidemiology (104, pp. 692–93), states that “[b]iological classification of human races is difficult—and sometimes meaningless—because of significant genetic and environmental overlaps among population groups. Concepts of race often reflect social and ideological conventions.” However, it continues, “Socioeconomic, cultural, and behavioral differences are often more important than racial differences in influencing health status,” which begs the question, what then are “racial” differences, if not socioeconomic, cultural, and, we would add, sociopolitical and environmental? It resumes, “However, race may be a useful concept in public health because some exposures and diseases are correlated with biological and physical aspects of race” (pp. 692–93), suggesting that the public health utility of race is biological. Although the dictionary acknowledges that the public health significance of the biological and physical aspects of race “may relate to gene-environment interactions or to specific gene variants, which may be associated with environmental exposures of prior generations,” it contends that “[u]seful insights into human biology and genetics have come from analysis by racial group” (pp. 692–93). Therein lies the problem in both seeking and interpreting the (independent) race effect.

As described above, historical evidence indicates that the evolution of racial classification in the United States grew out of a systematic process of racial stratification and objectification that was motivated by economic interests and resulted in a race-based class structure with Blacks as (forced) laborers and Europeans as owners (96, 128). This race-based class structure endured postemancipation, reconstruction, and the backlash of Jim Crow; despite the gains in civil rights, this structure continues to this day, as evidenced by racial differences in the distribution of SEP. Although not legally enforced, the enduring racially motivated class structure in the United States reified arguments about the inferiority of some groups relative to others and continues to find a home in contemporary practices and norms such as labor and wage discrimination (123); workplace discrimination, including organizational behavior related to decision making, and informal networks in relation to mentoring and advancement, which can lead to what Pager & Shepherd call “homosocial reproduction” (98, p. 16); housing and lending (98); educational policy and practice, including admissions, inclusive versus exclusive classroom practices, and teacher prejudice (52); and race-based consumer marketing (98). These norms and practices contribute to the intergenerational transmission of (dis)advantage or what Oliver & Shapiro (100) refer to as the “sedimentation of racial inequality” (pp. 5, 52–54).

Given this history, there are inherent challenges associated with estimating an independent race effect. For example, it is not uncommon to find authors interpreting the residual race effect after accounting for various measures of SEP and behavioral and health system factors as evidence for genetic differences between racial groups (18). Such practices reflect perhaps a particular, even if implicit, ontological frame or a procedural decontextualizing of race that ignores unmeasured confounding by a vast array of social-environmental factors. A hotly debated study by Van den Oord & Rowe (116) found that racial differences in birth weight were explained by environmental rather than genetic factors. However, the study authors interpreted the findings as potentially due to unmeasured genetic factors, demonstrating how subjectivity (i.e., philosophical assumptions and interpretive frames), explicit or implicit, enters the scientific process despite claims of “strong objectivity” (47). Even authors examining racial admixture as a measure of biological race cite evidence of significant admixture among ethnic populations (as opposed to between) and concede that racial admixture is “likely correlated with a range of social, cultural, and/or environmental variables that influence disease occurrence yet remain unmeasured,” that “SES is associated with genetic ancestry leading to confounding in tests for individual markers,” and that “nongenetic factors may account for all or part of the association between a phenotype and ancestry” (105, pp. 474–75). In his seminal paper, “The Contribution of the Social Environment to Host Resistance,” Cassel (22) states,

“Epidemiology at any given time is something more than the total of its established facts. It includes their orderly arrangement into chains of inference which extend beyond the bounds of direct observation.” It is this “orderly arrangement into chains of inference” which intrigues me and which I think distinguishes creative epidemiologic studies from studies which may display considerable rigor in their methods but which are essentially pedestrian. The question then is, what guides us in developing these chains of inference? Unquestionably, in large part the answer is the model of disease causation which we (implicitly or explicitly) espouse. (p. 107)

Thus, there is a need for philosophical rigor—making explicit our assumptions and interpretive frames—in our efforts to contextualize and hence justify our research questions, methodological approaches, and interpretation of results. How we conceptualize not only race but also racial health inequalities (e.g., social versus biological, inequalities versus inequities) determines the questions we ask, what we measure, how we measure it and among whom, and the validity of our conclusions (i.e., how close we come to the truth). This argument is equally important in studies of SEP inequalities. One challenge limiting philosophical rigor in studies of racial and SEP inequalities is the conventional search for causal effects using the “effects-of-causesapproach” (i.e., what is the effect of cause A?) without consideration of causal theory, the “causes-of-effects approach” (i.e., what is the cause of outcome Y?) (82, p. 230). Zuberi notes, “A causal effect is the effect of a factor on a given response variable, whereas causal theories consider how and why the effect operates...the causal theory serves a more fundamental purpose in social statistics” (128, p. 125). Considering race, one might ponder whether it is more useful to examine the “effects of race” (117), the average effect of R on Y, or to ask a fundamentally different question: Why is race reliable in predicting Y? (i.e., what processes are responsible for the “effects of race”)? The latter is a question of fundamental causes, of historical processes, of racial formation, and of the very concept of race itself, minimally leading us to measure social processes themselves and to determine how best to measure those processes rather than using race as a proxy for innumerable unconfined interpretations. Alternatively, and perhaps ultimately, these interrogations call for what Breihl refers to as a more “critical (social) epidemiology” (19).

In asking what is the relative role of race and SEP, are we to intervene on race itself or on the factors that maintain racial differences in health? Heathy People 2010 had the goal of eliminating health inequalities and ensuring optimal health for all (91). Although great strides have been made in improving population health, there has been little progress in reducing the health gap between groups, suggesting that the causes of population health are not the same as the causes of health inequalities. Frolich & Potvin (39) describe how the flexible nature of material and nonmaterial (e.g., social connections) resources may result in differential intervention effects and advocate for focusing on socially defined subpopulations that have a higher mean distribution of risk, what Phelan & Link call “risk of risks” (102, p. S30). To this end, Healthy People 2020 added social determinants to its list of Leading Health Indicators (26). It is generally well accepted that the social determinants of health are not randomly distributed. Hence, (social) epidemiology may be redefined as (*a*) the study of the population distribution of the (social) determinants of health and how that distribution impacts the distribution of health and illness within and across populations, and (*b*) application of this study to improving population health and remediating (tackle, face up to, challenge, threaten) health inequalities and health inequities. If our goal is to address both population health and health inequities, interrogating current epidemiologic practices with the aim of improving our scientific inquiry is critical.

## 4. CONCLUSION

A vast literature has examined health inequalities by race and SEP. However, conceptual and methodological challenges complicate the interpretation of study findings. Fundamental to these challenges is a lack of clarity about what race is and what that means for identifying study questions and hypotheses, study design characteristics, variable selection and specification, selecting an analytic strategy, and interpreting study findings. Additionally, there is wide variability in how SEP is conceptualized and measured, resulting in a lack of comparability across studies and significant misclassification of risk. A more careful examination of the causes of racial and socioeconomic health inequalities will inform efforts to improve population health and reduce health inequities. Intersectionality holds promise as both a theoretical and a methodologic orientation and requires consideration of the historical and contemporary context that determines the vector of resources and risk factors to which people are exposed on a day-to-day basis, impacting their interactions with the social world around them and, consequently, their psychology, their behavior, and their biology. Along these lines, further examination of how place becomes the site of intersection for race and SEP may help inform structural-level interventions that go beyond the individual to understand health-associated risk and resilience.

Additionally, although we have focused on the US context and primarily on Blacks and Whites, the issues raised here have implications for other racial and ethnic groups and other geographies contending with racial and SEP inequalities in health (6, 44, 114).

## Supplementary Material

supplemental

## Figures and Tables

**Table 1:** Median net worth and income quintile by race/ethnicity, 2000. Adapted from Reference 79, *Journal of Urban Health* © 2005; 82:iii26–iii34. LaVeist TA. Disentangling race and socioeconomic status: a key to understanding health inequalities. Published by Springer. Printed with permission. All rights reserved

Income quintile	Black	White	Hispanic
Lowest 20%	<$100	$24,000	$500
Second 20%	$5,275	$48,500	$5,670
Middle 20%	$11,500	$59,500	$11,200
Fourth 20%	$32,600	$98,842	$36,225
Highest 20%	$65,141	$208,023	$73,032

## SOCIAL STATISTICS

Since the time of Adolphe Quetlet (c. 1823), the first social statistician, the “average” man has been a primary focus of statistical investigation. Many statisticians who came after Quetlet (e.g., Galton, Edgeworth, Pearson), under the pretense of understanding human variation by identifying subpopulations within the so-called bell curve, emphasized mean values as a way to distinguish between or categorize social groups—distinguishing between groups defined by class and race to argue for the inheritance of genius—at once marking the beginning of eugenics and setting the tone for social statistics for years to come. One early and influential example is Yule’s work (1895–1899) examining poverty and poor relief in the United Kingdom. Extending the theories of Galton and Pearson, though not endorsing eugenics, Yule’s analysis is an early example of applying these ideas to statistics for the purpose of social analysis. He notes, “High average values of the former correspond to higher average values of the latter” (87, p. 605). Further elaborating on these ideas, his theory of estimation integrating multiple regression and least squares theory, basing inference on mean values of *x* and *y*, is lauded as one of the most influential theories in the modern field of quantitative social science.

## THE DIMINISHING RETURNS HYPOTHESIS

The diminishing returns hypothesis states that African Americans experience diminishing health returns for increasing investments in SEP. This hypothesis is corroborated by evidence demonstrating that significant racial health disparities exist at very high levels of income and education. High SEP does not buy the same level of health for African Americans relative to Whites. One potential explanation for this observation is the high level of racism-related stress that many African Americans face as they climb the social ladder. Reasons for this include stereotype threat; increased exposure to all-White or mostly White spaces (e.g., work environment), resulting in both stereotype threat and tokenism; as well as patterns of appraisal and coping that sometimes accompany social gains for African Americans, such as John Henryism and Superwoman Schema. These patterns of coping may be particularly important for higher SEP racial minorities contending with the social realities of interacting in predominantly White spaces. However, studies show that regardless of income, African Americans have a higher predicted probability of allostatic load and faster rate of telomere attrition than do their White counterparts.
