# Supplementary material for: Relative Roles of Race Versus Socioeconomic Position in Studies of Health Inequalities: A Matter of Interpretation
Source: Annu Rev Public Health. Author manuscript; Available in PMC 2023 Mar 27. (PMC10042447; doi:10.1146/annurev-publhealth-040617-014230)
Supplement: supplemental [file NIHMS1876758-supplement-supplemental.pdf]

Supplemental Material: Annu. Rev. Public Health 2018. 39:169–88
https://doi.org/10.1146/annurev-publhealth-040617-014230
Relative Roles of Race Versus Socioeconomic Position in Studies of Health Inequalities: A Matter of Interpretation
Nuru-Jeter et al.

Supplemental Figure 1. *American Journal of Epidemiology* citations by discipline. From *American Journal of Epidemiology* © 2008; 161(5):494-500. Oakes JM. An analysis of *American Journal of Epidemiology* citations with special reference to statistics and social science. Published by Oxford University Press. Printed with permission. All rights reserved.

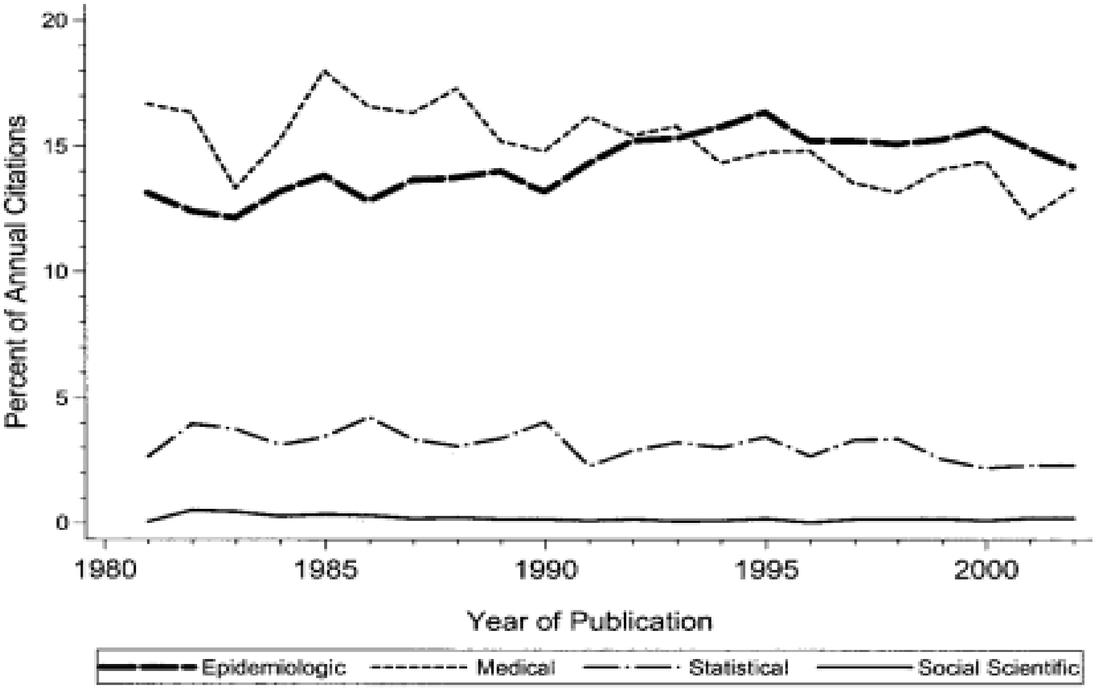

Supplemental Figure 2 Socioeconomic position over the life course. From *British Medical Bulletin* © 2007 (1). Galobardes B, Lynch J, Smith GD. Measuring socioeconomic position in health research. Published by Oxford University Press. Printed with permission. All rights reserved.

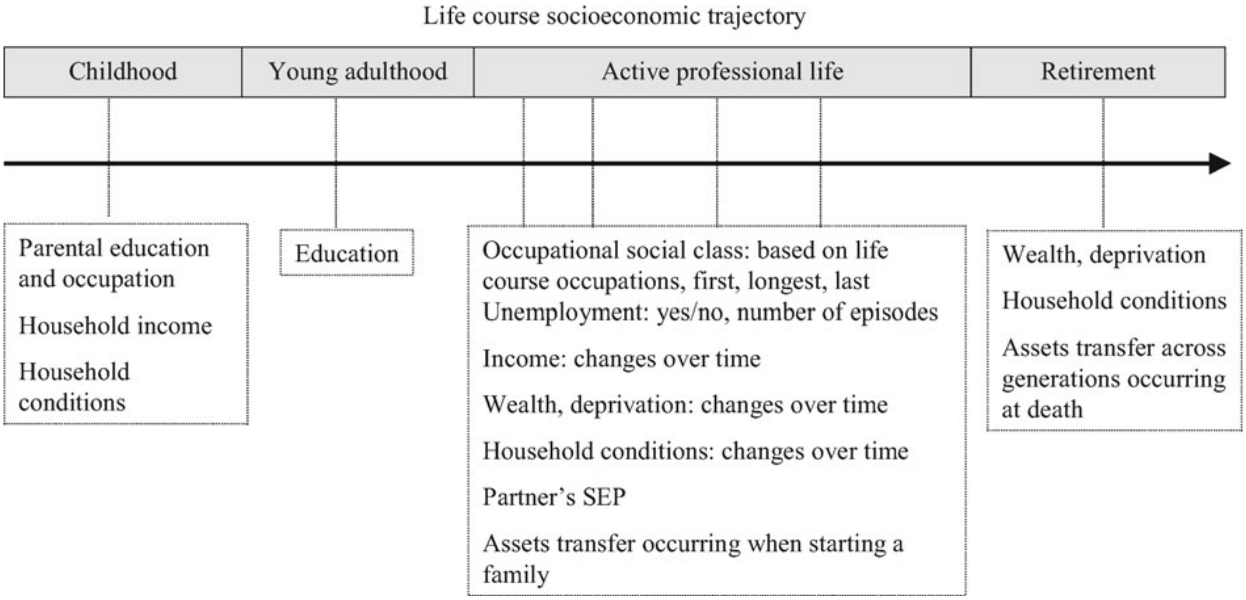

Supplemental Figure 3 Direct and indirect pathway between race and disease. From *American Journal of Epidemiology* © 2001;154(4):291-8. Kaufman JS, Cooper RS. Commentary: considerations for use of racial/ethnic classification in etiologic research. Published by Oxford University Press. Printed with permission. All rights reserved.

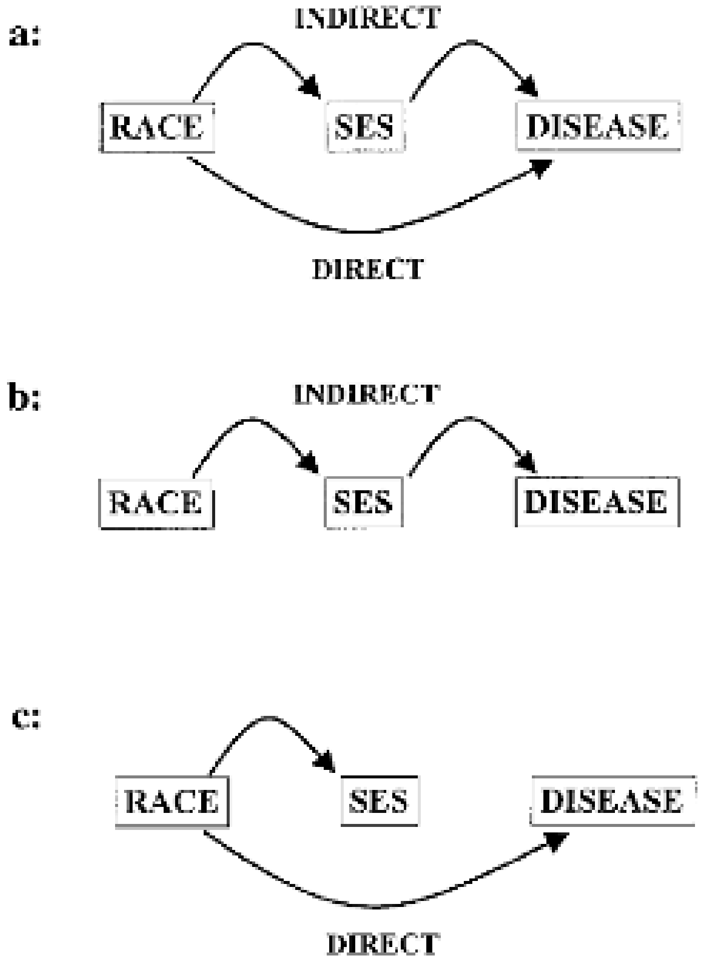

Supplemental Figure 4 Model of racism as a fundamental cause. Reproduced with permission of *Annual Review of Sociology*, Volume 41:311-30 in 2015 © by Annual Reviews, http://www.annualreviews.org

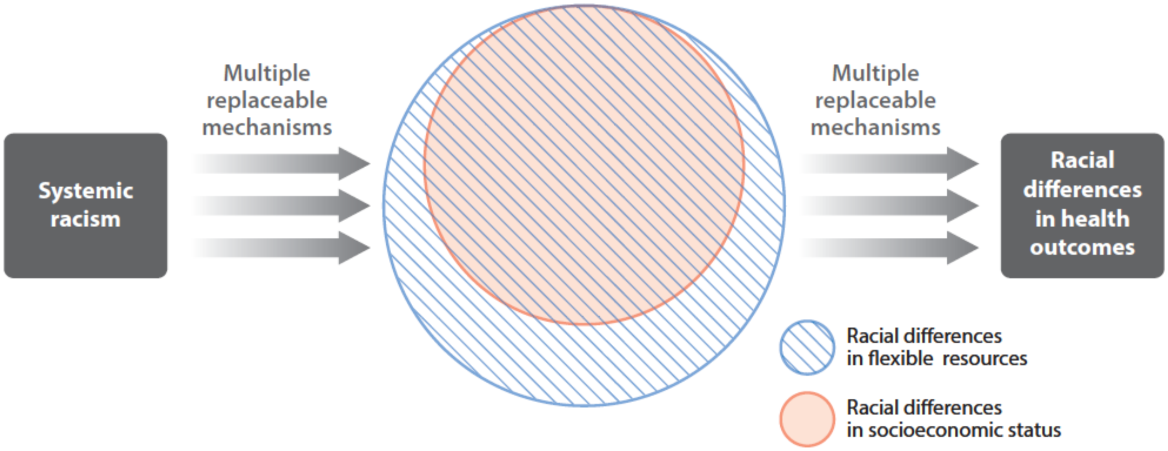

Supplemental Figure 5 General health status, by self-identified and socially assigned "race." From *Ethnicity & Disease* © 2008. Jones, Camara Phyllis et al. Published by ISHIB. Printed with permission. All rights reserved.

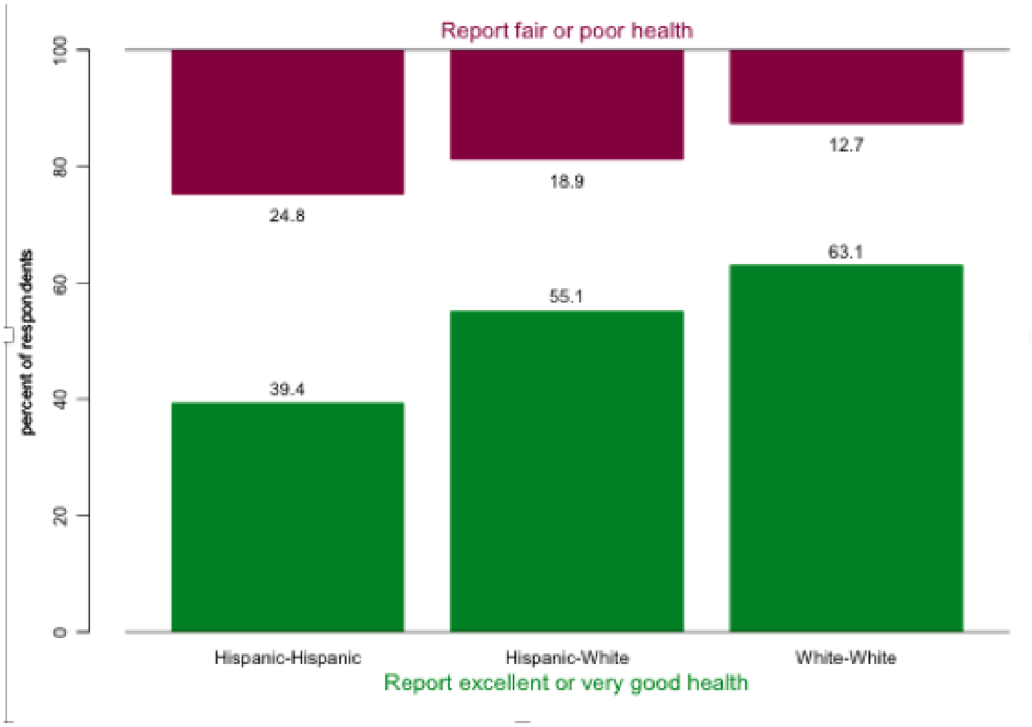

Supplemental Figure 6 Percent of population whose general health status is excellent or very good by self-identified race/ethnicity and socially assigned race. From *Ethnicity & Disease* © 2008. Jones, Camara Phyllis et al. Published by ISHIB. Printed with permission. All rights reserved.

	Socially assigned race							
Self-identified "race"/ethnicity	White	Black	Hispanic	Am Indian	Asian	NHOPI	Other	Row marginals
White								
% excellent or very good	58.6		50.3				49.2	58.4
95% confidence interval	*57.8–59.5*		*36.1–64.4*				*34.6–63.9*	*57.6–59.3*
Black								
% excellent or very good		44.3						44.0
95% confidence interval		*42.3–46.2*						*42.1–45.9*
Hispanic								
% excellent or very good	53.7	44.4	39.8					43.7
95% confidence interval	*46.2–60.9*	*28.9–61.2*	*35.3–44.6*					*40.0–47.4*
American Indian								
% excellent or very good	52.6			32.0				42.4
95% confidence interval	*41.3–63.8*			*21.8–44.3*				*34.7–50.5*
Asian								
% excellent or very good					60.6			62.6
95% confidence interval					*49.7–70.5*			*53.2–71.1*
NHOPI								
% excellent or very good								
95% confidence interval								
Other								
% excellent or very good	50.4							45.6
95% confidence interval	*35.8–64.9*							*36.1–55.5*
More than one race								
% excellent or very good	53.5	30.7						45.7
95% confidence interval	*44.3–62.5*	*19.7–44.4*						*38.6–53.0*

Supplemental Figure 7 Physiognomy model. From *Health Services Research* © 1994;29(1):1. LaVeist TA. Beyond dummy variables and sample selection: what health services researchers ought to know about race as a variable. Published by John Wiley and Sons. All rights reserved.

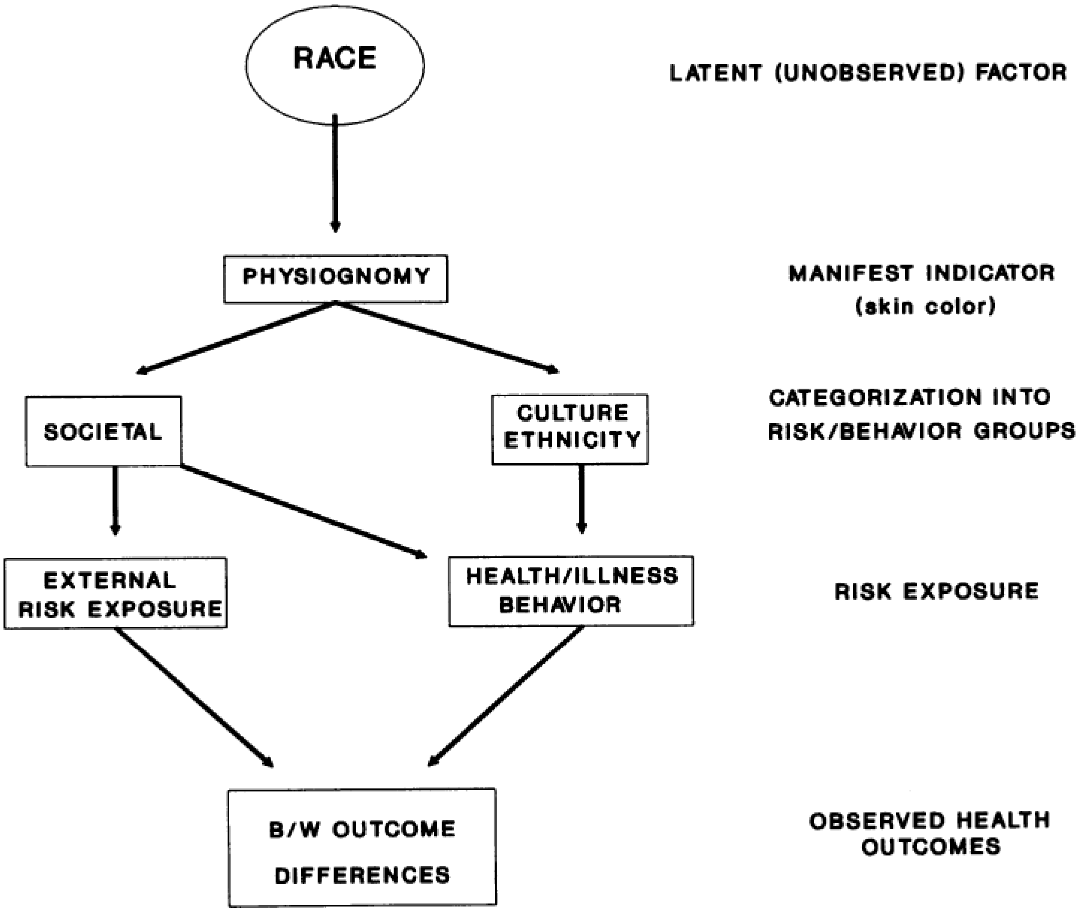

Supplemental Material: Annu. Rev. Public Health 2018. 39:169–88
https://doi.org/10.1146/annurev-publhealth-040617-014230
Relative Roles of Race Versus Socioeconomic Position in Studies of Health Inequalities: A Matter of Interpretation
Nuru-Jeter et al.

Supplemental Table 1 Nested multivariate regression of psychological distress on race for full sample (n = 3587). From *Stress and Health* © 2008. Nuru-Jeter, Amani et al. Published by John Wiley and Sons. Printed with permission. All rights reserved.

	Model 1: race		Model 2: income		Model 3: gender		Model 4: race × income†		Model 5: education and health problems		Model 6: marital status and age	
	β	SE	β	SE	β	SE	β	SE	β	SE	β	ED
Black	0.32*	0.19	0.06	0.19	0.04	0.19	0.80*	0.44	0.71	0.44	0.52	0.43
Hispanic	0.58***	0.19	0.32	0.19	0.31	0.19	0.64	0.47	0.92**	0.46	0.55	0.46
API	0.24	0.22	0.12	0.22	0.09	0.22	−0.53	0.55	−0.05	0.54	−0.14	0.54
Income	—	—	−0.44****	0.04	−0.41****	0.04	−0.36****	0.07	−0.23****	0.07	−0.17***	0.07
Household size	—	—	0.13**	0.05	0.13***	0.05	0.13***	0.05	0.15***	0.05	0.12**	0.06
Gender	—	—	—	—	0.84****	0.14	0.83****	0.14	0.79****	0.14	0.78***	0.14
Black × Income	—	—	—	—	—	—	−0.19**	0.10	−0.16	0.10	−0.16*	0.10
Hispanic × Income	—	—	—	—	—	—	−0.08	0.10	−0.12	0.10	−0.11	0.10
API × Income	—	—	—	—	—	—	0.14	0.11	0.08	0.11	0.05	0.11
Education	—	—	—	—	—	—	—	—	−0.18***	0.06	−0.24****	0.06
Health problems	—	—	—	—	—	—	—	—	2.20****	0.21	2.38****	0.21
Marital status	—	—	—	—	—	—	—	—	—	—	0.71****	0.16
Age	—	—	—	—	—	—	—	—	—	—	−0.03****	0.00
Adjusted R^2												0.10

*$p < 0.10$; **$p < 0.05$; ***$p < 0.01$; ****$p < 0.001$.
Black (1 = yes, 2 = no); Hispanic (1 = yes, 2 = no); API (1 = yes, 2 = no); gender (1 = women, 2 = men); health problems (1 = yes, 0 = no); marital status (1 = not married, 0 = married).
API: Asian/Pacific Islander; SE: standard error.
† [Correction made here after initial online publication]
